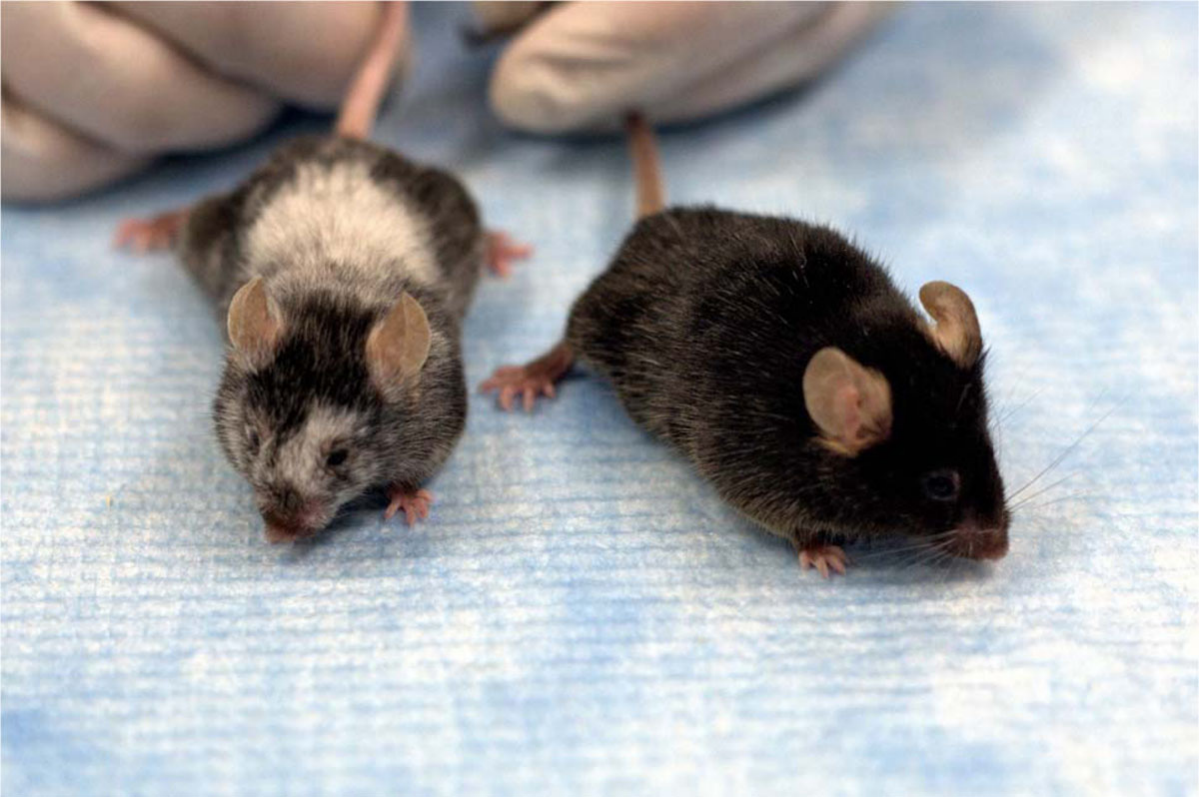# Modulating immunometabolism of tumor specific mouse and human lymphocytes to enhance T cell based therapy for cancer

**DOI:** 10.1186/2051-1426-3-S2-P325

**Published:** 2015-11-04

**Authors:** Madhusudhanan Sukumar, Jie Liu, Shashank J Patel, Christopher A Klebanoff, Gautam Mehta, Rahul Roychoudhuri, Joseph Crompton, David Clever, Luca Gattinoni, Pawel Muranski, Toren Finkel, Nicholas Restifo

**Affiliations:** 1 Center for Cancer Research, NCI/NIH, Bethesda, MD, USA; 2 National Institutes of Health, Rockville, MD, USA; 3 Center for Cancer Research, NCI/NIH, Rockville, MD, USA; 4 Experimental Transplantation and Immunology Branch, NCI/NIH, Bethesda, MD, USA; 5 NIH, Bethesda, MD, USA

## 

Tumor cells and tumor infiltrating lymphocytes (TIL) competes for glucose and other metabolites within the tumor microenvironment for their survival. Glucose consumption by tumors metabolically restricts T cell's ability to produce effector cytokines and therefore approaches to improve the overall metabolic fitness of TIL may improve tumor regression in cancer patients. Long-term survival and anti-tumor immunity critically depends on their metabolic fitness but approaches to select metabolically robust T cells for adoptive immunotherapy remains less clear. Here we introduce a simple and clinically translatable method using a lipophilic cationic dye (tetramethylrhodamine methyl ester-TMRM) to identify and isolate metabolically-robust T cells based on mitochondrial membrane-potential (∆Ψm). Cells with lower membrane-potential (low-∆Ψm) had a molecular profile characteristic of memory precursors and displayed an enhanced ability to enter the memory pool as compared to cells displaying higher mitochondrial potential (high-∆Ψm) characteristic of short-lived effectors. Interestingly, we also found that multiple distinct negative inhibitory receptors such as programmed death-1 (PD-1), cytotoxic T-lymphocyte-associated protein 4 (CTLA-4), B- and T-lymphocyte attenuator (BTLA), Lymphocyte-activation gene 3 (LAG-3) and T cell immunoglobulin mucin receptor 3 (TIM-3) were enriched in the high-∆Ψm subset compared to the low-∆Ψm subset. Comprehensive metabolomic and gene expression profiling demonstrated global features of metabolic fitness in low ∆Ψm sorted CD8^+^ T cells—including reduced glycolysis, enhanced fatty-acid oxidation and robust spare respiratory capacity. Transfer of low-∆Ψm T cells was associated with superior long-term in vivo persistence as evidenced by 100 fold increase in the frequency of T cells 300 days after adoptive transfer, augmented autoimmunity and an enhanced capacity to eradicate established cancer compared with high-∆Ψm cells. High-∆Ψm T cells exhibited elevated ROS levels, increased effector cytokines and had up-regulation of genes involved in DNA replication, DNA repair and cell-cycle arrest genes compared to low-∆Ψm T cells. Surprisingly, use of Ψm to enrich for cells with superior metabolic features was observed within central-memory (T_CM_) and effector (Tc17, Th1, Th17) T cells as well as long-term hematopoietic stem cells (LT-HSC). Finally, we also demonstrate that mitochondrial membrane potential based sorting can identify CD45RO^-^ CCR7^+^ human CD8^+^ T cells. These findings demonstrate that metabolic-sorting serves as a complementary strategy to the use of conventional cell surface markers for identifying cells with the capacity for long-term survival and ongoing effector function after adoptive-transfer. This novel metabolism-based approach may be broadly applicable to therapies involving transfer of hematopoietic stem cells or lymphocytes for treatment of viral-associated illnesses and advanced cancer.

**Figure 1:**